# The Strengthening of Quadriceps, Abductors, and External Rotator Muscles of the Hip to Alter Axial Alignment of the Lower Limbs in University Students with Patellofemoral Pain Syndrome: A Prospective Cohort Study

**DOI:** 10.3390/jfmk11020225

**Published:** 2026-06-01

**Authors:** Raphael Augusto Gir de Carvalho, Bianca Benelli Pizzolato, Guilherme Pasqualin Afonso de Souza, Evanil Minussi Filho, Gustavo Fonseca Lemos Calixto, Ewerton Alexandre Galdeano, Mariana Mattar Sampaio Madureira, Waldinei Merces Rodrigues, Marcelo Rodrigues da Cunha, Eduardo Gomes Machado, Fernando Bento Cunha, Rogerio Leone Buchaim, Marcelo de Azevedo Souza Munhoz

**Affiliations:** 1 Department of Graduate Studies in Health Sciences, Faculty of Medicine of Jundiaí (FMJ), Jundiaí 13202-550, Brazil; ra2201016@g.fmj.br (R.A.G.d.C.); ra2001107@g.fmj.br (B.B.P.); ra2001172@g.fmj.br (G.P.A.d.S.); evanilminussifilho@hotmail.com (E.M.F.); gustavo_calixto@hotmail.com (G.F.L.C.); educacaopermanente@hsvicente.org.br (E.A.G.); marimattarsampaio@gmail.com (M.M.S.M.); waldineirodrigues@g.fmj.br (W.M.R.); marcelocunha@g.fmj.br (M.R.d.C.); eduardomachado@g.fmj.br (E.G.M.); fernandocunha@g.fmj.br (F.B.C.); marcelomunhoz@g.fmj.br (M.d.A.S.M.); 2 Department of Teaching and Research, São Vicente de Paulo Charity Hospital, Jundiaí 13201-625, Brazil; 3 Department of Biological Sciences, Bauru School of Dentistry (FOB), University of Sao Paulo (USP), Bauru 17012-901, Brazil; 4 Center for the Study of Venoms and Venomous Animals (CEVAP), Sao Paulo State University (UNESP), Botucatu 18610-307, Brazil

**Keywords:** muscle strengthening, patellofemoral pain, femoral anteversion, tibial tubercle-trochlear groove distance, tibial torsion

## Abstract

**Background**: Proximal lower-extremity muscle strengthening is an important conservative intervention for patellofemoral pain syndrome (PFPS), as these muscle groups play critical roles in femoral stabilization and knee valgus control. However, evidence remains limited regarding the effectiveness of muscle strengthening in improving lower-extremity axial alignment through modulation of femoral neck anteversion, femoral internal rotation, and tibial external rotation. Therefore, the present study aimed to determine whether a strengthening protocol targeting the quadriceps and hip external rotator and hip abductor muscles could improve knee alignment and reduce bone torsion in young adults with patellofemoral pain syndrome. **Methods**: This prospective interventional cohort study implemented a muscle strengthening protocol in ten university students with PFPS. Outcomes included femoral neck anteversion angle (FNA), tibial tubercle–trochlear groove distance (TT–TG), tibial external torsion angle (TET), and the knee Q-angle, assessed via 3D reconstruction of computed tomography (3D-CT) images. Pre- and post-intervention data were analyzed using the Shapiro-Wilk test for normality and repeated-measures ANOVA (*p* < 0.05; 95% confidence interval). **Results**: Muscle strengthening improved lower-limb axial alignment, with reductions observed across all measures post-intervention. Mean changes were 0.68 ± 1.26° for FNA (*p* = 0.0626); 1.51 ± 0.97 mm for TT–TG (*p* = 0.0001); 1.38 ± 3.36° for TET (*p* = 0.2231); and 1.14 ± 1.52° for the Q-angle. Statistically significant improvements were observed for TT–TG and the Q-angle. **Conclusions**: Proximal muscle strengthening improved knee valgus and axial lower-limb alignment, as evidenced by significant reductions in Q angle and TT–TG distance. Reductions in femoral neck anteversion (FNA) and tibial external torsion angle (TET) were observed. However, these differences were not statistically significant. These findings support muscle strengthening as a noninvasive strategy for improving lower-limb alignment in individuals with patellofemoral pain syndrome.

## 1. Introduction

Patellofemoral pain syndrome (PFPS) is among the most common causes of anterior knee pain, particularly affecting physically active individuals and young women. It accounts for a substantial proportion of musculoskeletal complaints managed in orthopedic and physical therapy settings, with an estimated prevalence ranging from 15% to 45% in athletic populations and approximately 22–30% among young adult women [1,2]. PFPS has a multifactorial etiology, encompassing anatomical, biomechanical, and functional components. Clinically, it is characterized by diffuse anterior knee pain and typically aggravated by activities that increase patellofemoral joint loading, such as stair negotiation, running, or prolonged sitting [3,4].

Several predisposing factors have been implicated in the development of PFPS, including patellar maltracking, dynamic knee valgus, weakness of the quadriceps and hip stabilizing musculature, and impaired neuromuscular control. Valgus malalignment, in particular, promotes lateral displacement of the patella during movement and has been identified as a significant contributor to excessive patellofemoral stress and persistent symptoms [5].

Management of PFPS may involve either surgical or conservative strategies, with non-surgical treatment recommended as the first-line approach in most cases [2]. Surgical procedures such as proximal or distal patellar realignment, lateral retinacular release, and tibial tubercle transfer are typically reserved for refractory cases, especially when marked structural malalignment is present or when conservative therapy fails to provide adequate symptom relief [6,7]. Conservative interventions include postural re-education, patellar taping, orthotic use, neuromuscular training, and structured muscle strengthening programs [7].

Within conservative management, muscle strengthening plays a pivotal role, particularly targeting the quadriceps, hip abductors, and external rotators. These muscle groups are essential for joint stabilization and controlling dynamic valgus and excessive femoral internal rotation during functional tasks [1,5,8]. Randomized controlled trials have demonstrated that strengthening protocols focusing on the hip and knee musculature effectively reduce pain and improve functional outcomes [9,10,11], enhance knee alignment as reflected by reductions in the Q angle [12], decrease joint loading [13], and increase muscle strength and activation capacity [14] following training periods of six to twelve weeks [9,12].

Knee alignment has traditionally been evaluated using the Q angle, defined by the intersection of lines drawn from the anterior superior iliac spine to the center of the patella and from the patella to the tibial tuberosity [15]. Despite its widespread clinical use, this measure has important limitations, as it is two-dimensional and highly dependent on examiner technique, hip positioning, and subject posture, which undermines its validity and reproducibility as an indicator of patellofemoral dysfunction [16,17].

More recently, advanced methods for assessing axial knee alignment have been described, particularly the measurement of the tibial tubercle–trochlear groove (TT–TG) distance using computed tomography or magnetic resonance imaging. This variable quantifies the lateral displacement of the tibial tuberosity relative to the trochlear groove and is widely employed to assess the severity of femorotibial malalignment. TT–TG values exceeding 20 mm are considered pathological and have been associated with increased knee valgus, patellar instability, and persistent anterior knee pain [18,19]. This imaging-based assessment demonstrates high reliability and reproducibility and offers superior sensitivity for detecting axial deviations that are not captured by the clinical Q angle [20].

Morphological and biomechanical variations at both proximal and distal segments of the lower limb may further influence knee alignment, including increased femoral neck anteversion angle (FNA) and tibial external torsion angle (TET). These rotational abnormalities affect the mechanical axis and patellar orientation, potentially exacerbating dynamic malalignment and intensifying patellofemoral symptoms [21,22,23,24].

Current evidence highlights a shift in non-surgical management toward interventions that extend beyond isolated muscle strengthening to include enhancement of neuromuscular control at the hip and knee. Functional rehabilitation programs aimed at promoting dynamic lower limb realignment have shown promising results [25,26]. Clinical studies suggest that strengthening of the quadriceps and proximal musculature can lead to reductions in the Q angle [27,28], while other investigations have reported significant decreases in dynamic knee valgus assessed through three-dimensional kinematic analyses [25,29].

With regard to strategies for assessing knee valgus, available methods can be broadly categorized into those based on static structural analysis and those designed for dynamic functional assessment. The literature consistently describes frontal-plane structural evaluation, typically performed using clinical goniometry. While this approach shows a significant correlation with radiographic measures, its reliability and validity remain variable across studies. In addition, it is limited to isolated assessment of the knee joint and does not account for proximal or distal morphostructural alterations of the lower limb [30].

In this context, full-length standing radiography represents a more comprehensive approach, as it enables evaluation of the global mechanical axis of the lower limb through the Hip–Knee–Ankle (HKA) angle. This measure is widely used in the planning of corrective procedures, such as osteotomies and arthroplasties, due to its greater precision in determining coronal alignment [31]. From a dynamic functional perspective, three-dimensional motion capture analysis constitutes a substantial advancement in orthopedics, as it allows identification of the contribution of muscle groups to knee behavior during movement [32]. However, these technologies predominantly focus on assessments in the frontal and sagittal planes and therefore present limitations in evaluating the rotational components of the femur and tibia, whose accurate quantification requires axial plane analysis [32,33].

To date, no study has investigated the effects of a specific and standardized muscle strengthening protocol in individuals with PFPS designed to promote improvement in knee alignment in the frontal plane and evaluate, in an integrated manner, the axial variables TT-TG, FNA, and TET, which are responsible for determining lower-limb bone torsion. In light of this gap, the present study aimed to determine whether a strengthening protocol targeting the quadriceps, hip external rotator, and hip abductor muscles is capable of correcting knee alignment and reducing bone torsion in young university students with patellofemoral pain syndrome.

## 2. Materials and Methods

This is a prospective interventional cohort study that evaluated the effects of a muscle strengthening program targeting the quadriceps femoris, hip abductors, and external rotators on lower limb axial alignment.

A total of 29 young adult participants, all university students enrolled at a medical school, were included in the study. Participants underwent two-dimensional and three-dimensional computed tomography assessments performed at a university hospital. Both the medical school and the hospital are located in the state of São Paulo, Brazil.

The study protocol was reviewed and approved by an institutional Research Ethics Committee—Research Ethics Committee on Human Subjects, Faculty of Medicine of Jundiai, Opinion Number: 3.890.549, approval date 28 February 2020. All procedures were conducted in accordance with the ethical principles for medical research involving human participants established by the Declaration of Helsinki. Written informed consent was obtained from all participants prior to enrollment in the study.

### 2.1. Eligibility

Participants aged 18 to 24 years were included if they met the following criteria: Physical fitness to perform physical activity, confirmed by clinical history and electrocardiography; reported femoropatellar pain during activities of daily living, such as squatting and stair climbing, rated as ≥5 on the Visual Analog Scale (VAS; 0–10 points); positive Clarke’s test for patellofemoral pain syndrome; availability to participate with the frequency required for the exercise program; increased or atypical knee Q angle, as determined by clinical examination using goniometry in the standing position. Angles >15° in men and >20° in women were considered atypical [34], as such patterns are associated with a higher risk of pain and patellofemoral malalignment [35].

When both knees exhibited similar pain intensity according to the VAS, only the knee with the greater valgus observed on clinical examination was selected for tomographic analysis. When both knees presented similar clinical Q angles, the knee with greater pain intensity according to the VAS was selected for analysis.

Demographic and anthropometric data, including sex, age, height, body weight, and body mass index (BMI), were collected before the intervention. Body weight and BMI were reassessed after completion of the training protocol in order to monitor possible anthropometric changes during the intervention period.

Participants were excluded from the study based on the following criteria: Refusal to provide informed consent; engagement in regular physical activity within the previous 12 months; pregnancy or lactation; history of oncological disease; systemic arterial hypertension; cardiac rhythm abnormalities identified by electrocardiography that contraindicated the performance of physical exercise; history of Osgood–Schlatter disease, slipped capital femoral epiphysis or ligament injuries; history of orthopedic surgical interventions involving the hip, knee, or ankle; absence from four or more physical training sessions (>10%); delay in performing the post-test computed tomography examination, with seven days considered the maximum allowable interval, calculated from the completion of the strengthening program to the execution of the examination.

The exclusion of participants who had engaged in regular physical activity during the 12 months preceding the intervention was intended to minimize potential biases related to sports training or exercise-induced mechanical overload. The diagnosis of patellofemoral pain syndrome was established through orthopedic and physical therapy clinical assessments based on clinical criteria previously described in the literature, including anterior knee pain associated with functional activities. Considering the multifactorial nature of patellofemoral pain syndrome, no specific etiological stratification of participants was performed, as the study was designed to analyze biomechanical alterations and lower-limb alignment.

### 2.2. Exercise Program for Muscle Strengthening

All participants were informed about the objectives of the treatment proposed by the study, as well as the required training frequency, which was established as three sessions per week over a twelve-week period, totaling thirty-six sessions. The exercises were performed either individually or in pairs at the university training center. The exercise sessions were conducted by the researchers and supervised by a licensed physical therapist, duly registered with the appropriate professional regulatory body, who was present during all training sessions.

Participants were previously evaluated by a physical therapist to determine the one-repetition maximum (1 RM) load for each exercise included in the muscle strengthening protocol. Training loads were individually prescribed based on the values obtained during the 1 RM assessment, according to established recommendations for progressive resistance training. During the intervention protocol, a recovery interval of 2 to 3 min was allowed between multi-joint exercises, in accordance with the recommendations of the American College of Sports Medicine for resistance training programs aimed at improving muscular strength [36].

**Figure 1:**
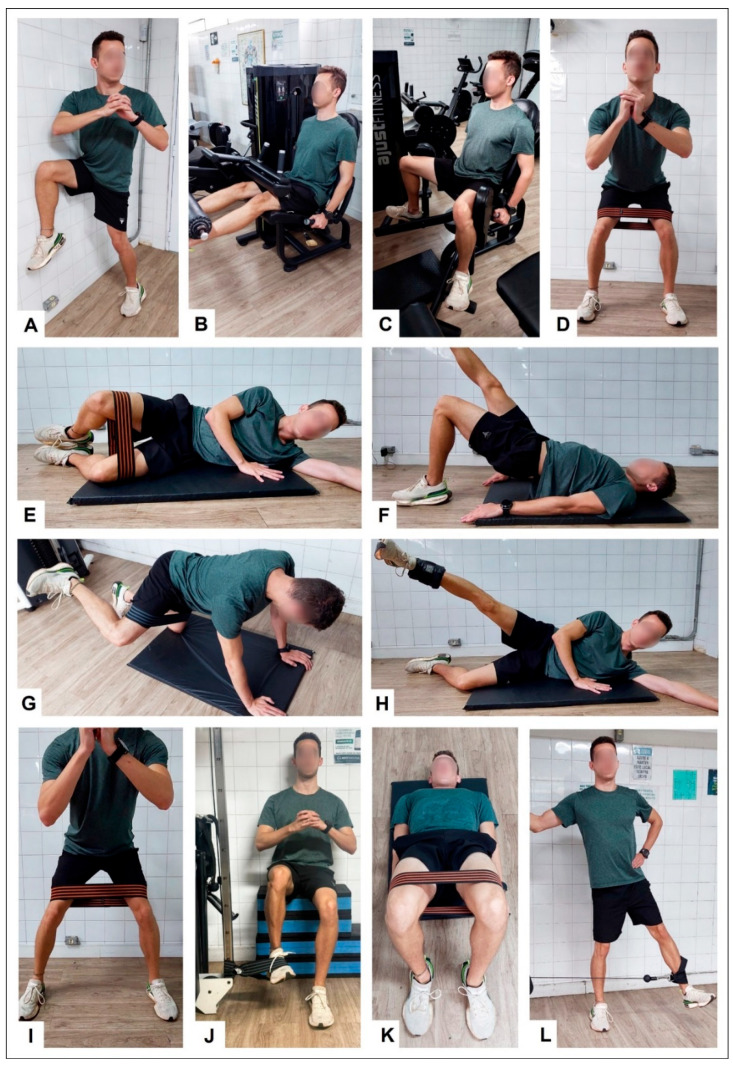
Photographic demonstration of the exercises performed in the strengthening program. (**A**) Isometric hip abduction exercise; (**B**) Knee extension exercise performed on a resistance machine with isotonic contraction; (**C**) Hip abduction exercise performed on an abductor machine with resisted isotonic contraction; (**D**) Squat with hip abduction and proprioceptive control; (**E**) Isotonic resistance exercise using a resistance band for hip abduction and external rotation; (**F**) Alternating single-leg hip bridge with isometric contraction; (**G**) Isotonic hip abduction exercise performed in the quadruped position with external rotation using resistance bands; (**H**) Hip abduction with leg extension using ankle resistance weights; (**I**) Lateral walk with band resistence; (**J**) Isotonic resistance exercise for external hip rotation using a pulley system in the seated position; (**K**) Hip bridge exercise with proprioceptive control during hip abduction using a resistance band; (**L**) Hip abduction combined with external hip rotation using a pulley system.

Each training session began with 5 min of stretching, followed by 15 min of treadmill walking at a speed of 4 to 5 km/h for muscle warm-up. Subsequently, participants underwent a strengthening program consisting of isometric and isotonic exercises targeting the quadriceps femoris muscles, external rotators, and hip abductor muscles. The strengthening program was designed to provide a sequence of exercises performed in three sets each, with a progressive increase in load, as detailed in Table 1 and illustrated in Figure 1.

As a complement to Table 1, Figure 1 presents photographic demonstrations of each exercise included in the strengthening program.

### 2.3. Computed Tomography (CT) Analyses

Two computed tomography examinations were performed: a baseline (control) scan and a post-intervention scan conducted after completion of the muscle strengthening program. Analyses were performed using computed tomography (CT) with a 1 mm slice thickness on a 32-channel Somatom Go.Up scanner manufactured by Siemens Healthineers^®^ (Erlangen, Germany).

Web Viewer^®^ version CT VA40 and Arya^®^ version 23.x software were used for two-dimensional (2D) image reconstruction for axial analyses of the femoral neck anteversion (FNA), tibial tuberosity–trochlear groove (TT–TG), and tibial external torsion (TET), as well as for three-dimensional (3D) image reconstruction for analysis of the Q angle. 

For the axial analyses, image superimposition was performed to assess the longitudinal axes of the femur and tibia, allowing identification of potential rotational alterations of the bony structures [37].

### 2.4. Positioning Criteria for Standardization of Imaging Examinations

Rotation of the limb during acquisition of axial images may influence results; therefore, proper alignment of the lower limb, including standardized positioning of the hip and knee, is recommended to minimize the risk of analytical error [38].

**Figure 2:**
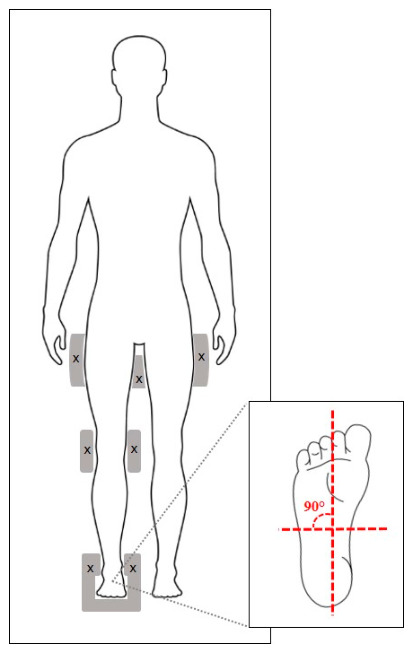
Standardization of participant positioning for computed tomography examination.

The same positioning protocol was applied for examinations performed before and after implementation of the exercise protocol. Participants were positioned in the supine position, with the upper limbs placed alongside the body. Foam supports were positioned at the hip, knee, and distally along the leg and foot to correct potential lower limb rotation during image acquisition. The image acquisition protocol aimed to position the limb under examination in a neutral alignment, with the foot oriented upward at 90° relative to the CT scanner table (Figure 2).

### 2.5. Analysis of the Femoral Neck Anteversion Angle (FNA)

**Figure 3:**
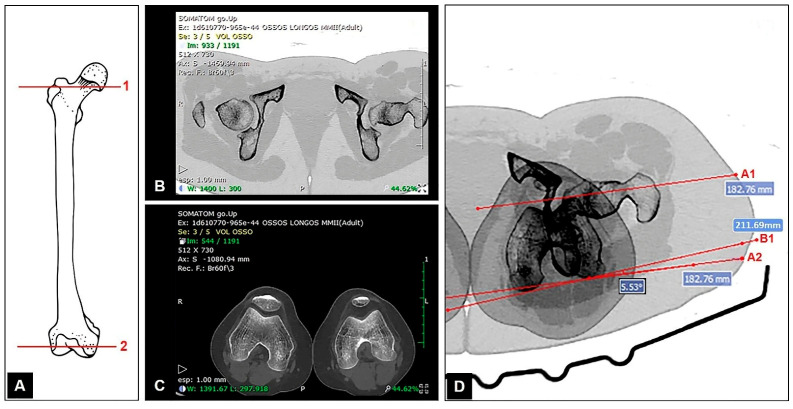
Analysis of the femoral neck anteversion angle (FNA) using computed tomography: (**A**) selected slices 1 and 2; (**B**) femoral neck slice (slice 1); (**C**) slice corresponding to the deepest point of the femoral trochlea (slice 2); (**D**) superimposition of axial images and FNA assessment (slices 1 and 2). (A1) longitudinal axis of the femoral neck; (B1) posterior transcondylar line; (A2) duplication of A1 positioned at its intersection with B1 to obtain the angle. The letters “R,” “L,” and “P” indicate laterality and anatomical position, representing right, left, and posterior, respectively.

The femoral neck anteversion angle (FNA) is defined as the angle formed between the axis of the femoral neck and a distal femoral reference, obtained through the superimposition of two CT images (Figure 3). As the standard analysis protocol for all study participants, the head–neck transition image (line 1 in Figure 3A) was selected, as this region provides optimal visualization of the femoral neck [39], resulting in the axial image shown in Figure 3B.

Subsequently, for the distal femoral reference, the slice corresponding to the deepest point of the trochlear groove was selected (line 2 in Figure 3A), yielding the tomographic image shown in Figure 3C. This image acquisition protocol allowed complete visualization of the posterior region of the femoral condyles, which were considered for FNA assessment.

After superimposition of the two images (Figure 3D), a line was drawn along the longitudinal axis of the femoral neck (A1). A transcondylar line was then drawn across the posterior aspects of the femoral condyles (B1). Finally, line A1 was duplicated (A2) and positioned at its intersection with line B1, thereby forming the femoral neck anteversion angle [40].

In the healthy adult population, the femoral neck anteversion angle (FNA) ranges from 8° to 20°, and values greater than 20° are considered increased [40]. A mean angle of 10.8° ± 8.7° has been observed in individuals without patellofemoral dysfunction, whereas a mean angle of 15.6° ± 9.0° has been identified in individuals with at least one episode of patellar dislocation [37].

### 2.6. Analysis of the Tibial Tubercle–Trochlear Groove (TT–TG) Distance

**Figure 4:**
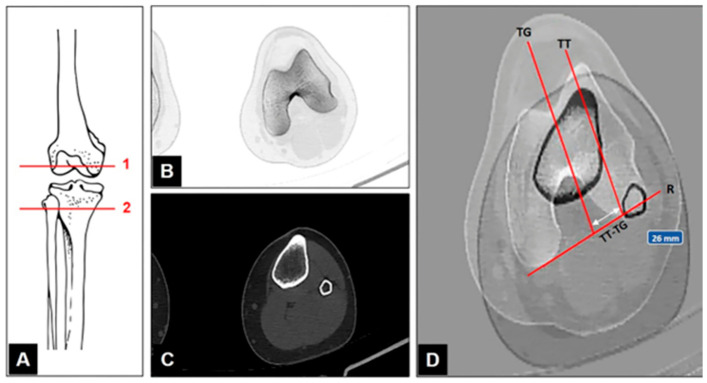
Analysis of the tibial tubercle–trochlear groove (TT–TG) distance using computed tomography: (**A**) selected slices 1 and 2; (**B**) slice corresponding to the deepest point of the femoral trochlea (slice 1); (**C**) slice of the apex of the tibial tubercle (slice 2); (**D**) superimposition of axial images and TT–TG assessment (slices 1 and 2). (R) posterior femoral transcondylar line; (TG) perpendicular line positioned at the deepest point of the femoral trochlea; (TT) perpendicular line positioned at the apex of the medial tibial tubercle.

The TT–TG distance is analyzed through the superimposition of two axial images that quantify the mediolateral displacement of the extensor mechanism (Figure 4). This displacement is measured in millimeters and corresponds to the distance between the apex of the tibial tubercle (TT) and the deepest point of the trochlear groove (TG), as illustrated by slices 1 and 2 in Figure 4A. The selected axial CT images are shown in Figure 4B,C.

After image superimposition, a reference line is drawn along the posterior aspects of the femoral condyles (R), and another line is positioned over the apex of the tibial tubercle (TT). Perpendicular to line R and parallel to the TT line, a third line is drawn through the deepest point of the trochlear groove (TG). The distance between the two parallel lines (TT–TG) is then measured in millimeters (mm), as demonstrated in Figure 4D. A TT–TG distance ≥ 20 mm is considered a pathological threshold and is frequently associated with patellar instability and an increased knee Q angle [18,19,37].

### 2.7. Analysis of the Tibial External Torsion Angle (TET)

**Figure 5:**
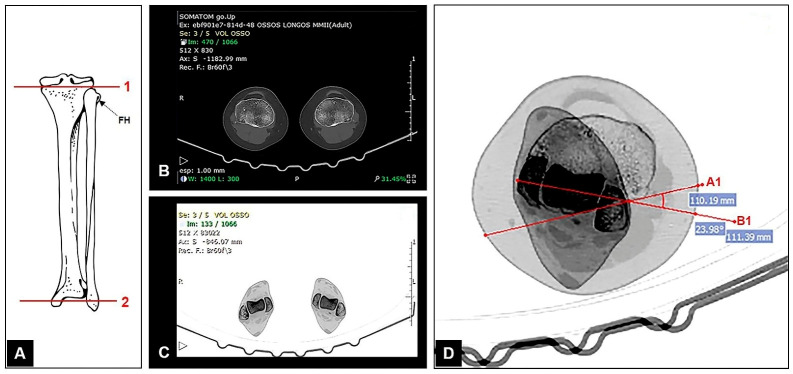
Analysis of the tibial external torsion angle (TET) using computed tomography: (**A**) selected slices 1 and 2; (**B**) tibial plateau slice (slice 1); (**C**) slice showing the greatest transmalleolar axis (slice 2); (**D**) superimposition of axial images and TET assessment (slices 1 and 2); (A1) line tangent to the posterior tibial plateau; (B1) line representing the transmalleolar axis. The letters “R,” “L,” and “P” indicate laterality and anatomical position, representing right, left, and posterior, respectively.

Axial images of the tibia were acquired, including one slice from the proximal region at the level of the tibial plateaus and another from the distal region at the transmalleolar level (Figure 5) [41]. Two additional criteria were applied to standardize the selection of baseline and follow-up slices:

(I) For the proximal region, the first slice immediately superior to the fibular head (FH) was selected, such that the FH was not visible on the image (Slice 1 in Figure 5A), resulting in the image shown in Figure 5B.
(II) For the distal region, the slice exhibiting the greatest transmalleolar axis was selected, measured in millimeters from the medial aspect of the tibial malleolus to the lateral aspect of the fibular malleolus (Slice 2 in Figure 5A), yielding the axial image shown in Figure 5C.

After superimposition of both images, a line tangent to the posterior tibial plateau (line A1) and another line along the transmalleolar axis (line B1) were drawn, forming an angle corresponding to the tibial external torsion angle, as illustrated in Figure 5D.

### 2.8. Analysis of the Q Angle Using Three-Dimensional Reconstruction (3D-CT)

**Figure 6:**
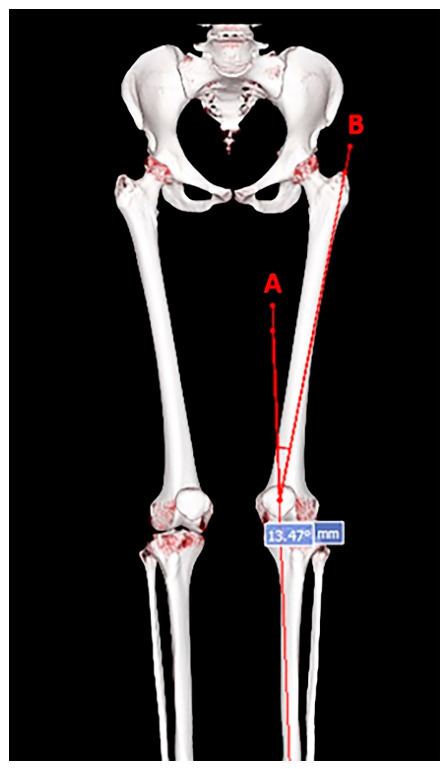
Q angle calculated from three-dimensional reconstruction of axial images of the lower limb. (**A**) Line drawn from the center of the patella to the tibial tubercle; (**B**) line drawn from the center of the patella to the greater trochanter of the femur.

The Q angle was assessed using three-dimensional axial reconstruction obtained by computed tomography (3D-CT). The analysis was performed in accordance with the evaluation protocol proposed by Lee and colleagues. One line was drawn from the apex of the greater trochanter of the femur to the center of the patella, and a second line was aligned from the center of the patella to the tibial tubercle; the intersection of these lines formed the Q angle [42], as shown in Figure 6.

### 2.9. Statistical Analysis

Data normality was assessed using the Shapiro–Wilk test. Statistical differences were analyzed using analysis of variance (ANOVA) for two related samples, comparing pre-test and post-test measurements.

All statistical analyses were performed using the IBM^®^ Statistical Package for the Social Sciences (SPSS), version 22. A significance level of *p* < 0.05 was adopted, and a 95% confidence interval was applied.

## 3. Results

The study was presented to a total population of 481 medical students, of whom 29 met the eligibility criteria and were enrolled in the muscle strengthening program. During the execution of the experiment, nineteen participants were excluded from the study for the following reasons: withdrawal from participation (*n* = 7); absence from more than 10% of the training sessions (*n* = 10); and delayed performance of the post-test computed tomography examination (*n* = 2). Consequently, data from 10 participants were included in the final analysis of this pilot study.

The sample consisted of six women and four men. Female participants presented a mean height of 1.66 m and a mean body mass index (BMI) of 22.12 kg/m^2^ before the exercise protocol, which decreased to 21.98 kg/m^2^ after the intervention. Male participants presented a mean height of 1.81 m, with a mean BMI of 27.2 kg/m^2^ before the intervention and 26.47 kg/m^2^ after completion of the training protocol.

The data were subjected to the Shapiro–Wilk normality test and demonstrated a normal distribution. Therefore, comparative analyses were performed using analysis of variance (ANOVA) for two related samples.

**Figure 7:**
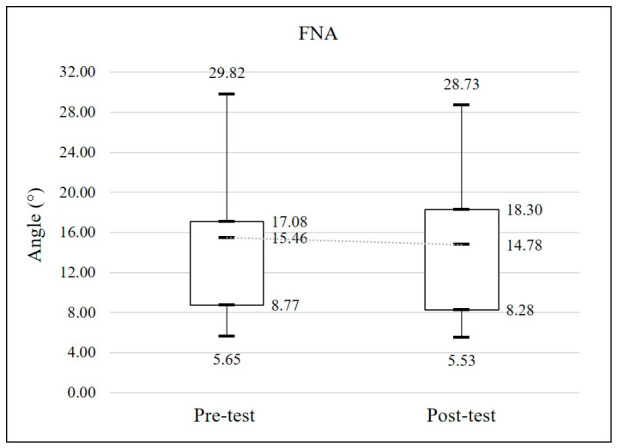
Graphical analysis of the femoral neck anteversion angle (FNA) results.

The femoral neck anteversion angle (FNA) showed a mean ± standard deviation of 15.46 ± 6.46° in the pre-test and 14.78 ± 8.29° in the post-test, representing a mean difference of 0.68 ± 1.26°, which was not statistically significant (*p* = 0.0626). The minimum and maximum values, quartiles, and means of the pre-test and post-test results are presented in Figure 7.

**Figure 8:**
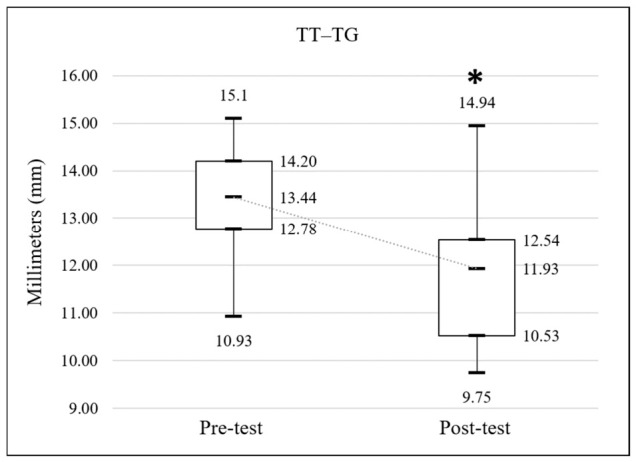
Graphical analysis of the tibial tubercle–trochlear groove (TT–TG) distance results. (*) Data set with a statistically significant difference.

The strengthening program targeting the quadriceps femoris muscles, hip abductors, and external rotators resulted in a significant reduction in the TT–TG distance (*p* = 0.0001). The TT–TG distance was, on average, 1.51 ± 0.97 mm lower compared with baseline values, as shown in Figure 8.

**Figure 9:**
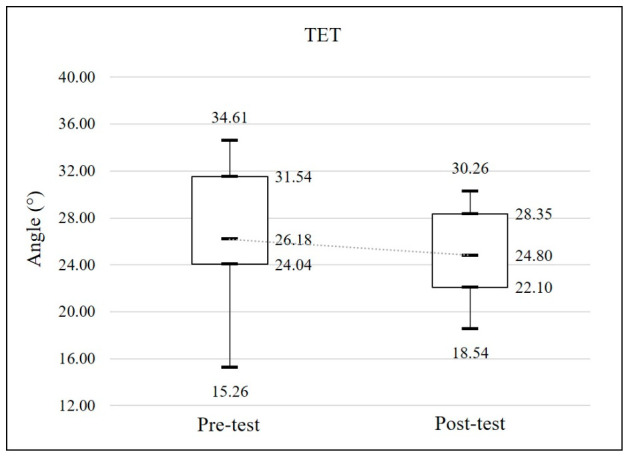
Graphical analysis of the tibial external torsion (TET) angle results.

No statistically significant difference was identified for TET (*p* = 0.2231). The TET showed a mean ± standard deviation of 26.18 ± 6.46° in the pre-test and 24.80 ± 4.12° in the post-test. The mean difference was 1.38 ± 3.36°, as illustrated in Figure 9.

**Figure 10:**
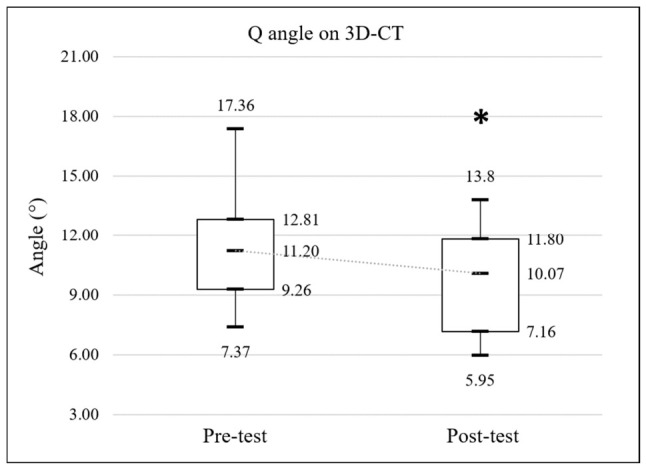
Graphical analysis of the knee Q angle results, assessed through three-dimensional axial reconstructions of computed tomography (3D-CT). (*) Data set with a statistically significant difference.

A significant reduction in knee valgus was observed, as confirmed by the Q angle measured on 3D-CT (*p* = 0.0091). The mean decrease was 1.14 ± 1.52° when comparing baseline data with measurements obtained after thirty-six training sessions. The results are presented in Figure 10.

## 4. Discussion

This pilot study demonstrated that the proposed muscle strengthening protocol was associated with significant reductions in the TT–TG distance and Q angle, as measured by three-dimensional computed tomography (3D-CT). In contrast, no significant changes were observed in the femoral neck anteversion angle (FNA) or the tibial external torsion angle (TET).

Despite the limitations of the pilot study design and the absence of a control group, the findings support the hypothesis that proximal muscle strengthening may contribute to improved knee alignment in both the frontal and axial planes. The literature consistently demonstrates that strengthening of the proximal musculature has positive effects on joint control during dynamic activities [25,32,43].

Strength training promotes neuromuscular adaptations capable of improving joint stability and biomechanical control of movement. Initial strength gains occur predominantly through neural mechanisms, including increased motor unit recruitment, higher neural firing frequency, improved muscle synchronization, and reduced antagonist coactivation, even before significant muscle hypertrophy develops [44]. These adaptations enhance motor control and neuromuscular coordination during functional activities, contributing to greater biomechanical efficiency and dynamic stability [5].

Furthermore, strengthening of the proximal stabilizing muscles of the hip and knee is associated with improved dynamic control of knee valgus, reducing compensatory movements and improving lower-extremity alignment during functional and sports-related tasks [13,25]. In this context, the present study provides preliminary evidence that muscle strengthening may also contribute to improvements in static lower-extremity alignment.

A potential mechanism underlying the observed findings may involve resting neurophysiological adaptations and increased resting muscle tone, which could partially explain the correction of static alignment observed in two of the variables evaluated in the present study. However, specific analyses would be required to further investigate these mechanisms, including electromyography and morphological assessments of the diameter, length, number, and structural characteristics of the quadriceps fibers, as well as of each muscle involved in hip abduction and external rotation.

According to the literature, resistance training promotes central neurophysiological adaptations associated with increased corticospinal excitability and improved motor unit recruitment, favoring enhanced neuromuscular control and biomechanical stability [45]. In addition, proximal strengthening and stabilization programs may increase muscle electromyographic activation, contributing to the reduction of dynamic knee valgus and improved functional alignment of the lower extremity [46]. Furthermore, strengthening exercises may modify resting muscle biomechanical properties, including muscle tone, muscle stiffness, and viscoelastic behavior, thereby contributing to greater joint stability and improved postural control following resistance training [47].

### 4.1. Functional Modifications Versus Structural Characteristics

The absence of changes in FNA and TET is consistent with the literature, which describes these variables as relatively stable in adulthood and not exhibiting measurable short-term alterations solely through muscle strengthening exercises [40]. Nevertheless, significant functional changes can occur in the absence of structural anatomical modifications. Recent biomechanical studies have shown that femorotibial alignment, particularly dynamic internal and external rotation, has a more immediate impact on patellofemoral mechanics than the bony version itself [48,49].

Additionally, individual differences in FNA may influence muscle activation patterns during hip exercises. Mitomo et al. demonstrated that women with increased femoral anteversion exhibit lower gluteus medius activation during the “clam” exercise at certain hip flexion angles [50]. While femoral anteversion is not altered by training, neuromuscular adaptations may optimize muscle recruitment patterns, thereby contributing to improved dynamic control and reduced patellofemoral joint overload [5,51].

### 4.2. Interpretation of the Reduction in TT–TG and Q Angle

The reduction in TT–TG is a particularly relevant finding, as elevated values are associated with lateral malalignment of the extensor mechanism and patellar instability [36,52]. Although TT–TG has traditionally been interpreted as a structural variable, recent evidence suggests that it may also be influenced by femorotibial rotation. Cadaveric studies have shown that each degree of internal or external knee rotation can alter the TT–TG distance by approximately 0.5 mm [53]. Therefore, improvements in proximal control, particularly through the reduction of excessive femoral internal rotation, may partially explain the decrease observed in the present study.

The concurrent behavior of the Q angle supports this interpretation. A significant correlation between Q angle and TT–TG has been demonstrated by Dickschas and colleagues [54]. Consequently, the simultaneous reduction of both measures suggests a common mechanism, likely related to increased dynamic hip stability, reduction of functional valgus, and improved biomechanical alignment of the lower limb.

### 4.3. Evidence for Hip Strengthening as a Modulator of Alignment

Several clinical trials have demonstrated significant benefits of strengthening the hip abductors and external rotators for improving pain, function, and dynamic knee alignment, particularly in individuals with patellofemoral pain syndrome (PFPS). A meta-analysis showed that hip-strengthening protocols were associated with greater reductions in pain (VAS) and improved function (Kujala/AKPS) compared with protocols focused exclusively on quadricep strengthening [55]. These findings are consistent with updated international guidelines for the conservative management of PFPS, which recommend hip exercises as a central component of treatment [1].

Furthermore, randomized clinical trials show that hip strengthening reduces pain more rapidly and improves control of dynamic valgus more effectively than programs centered solely on the knee [11,12]. Lee et al. demonstrated a significant reduction in dynamic Q angle and an improved VMO/VL activation ratio following functional exercises combined with hip strengthening [27], a finding closely aligned with the observations of the present study.

Additional results underscore the importance of selective proximal muscle strengthening in the treatment of patellofemoral dysfunction. A protocol targeting the gluteal muscles, hip abductors, and ankle dorsiflexors significantly reduced Q angle and knee pain compared with a protocol focused on quadriceps and ankle dorsiflexor strengthening [12]. A randomized controlled trial comparing exercise programs targeting proximal, distal, and local knee exercises in athletes with patellofemoral pain reported that the group performing proximal exercises showed significant reductions in pain and radiographic measures, such as the Q angle, patellar tilt angle, and congruence angle, suggesting positive effects on patellar realignment and symptom relief [28].

Collectively, these findings support the hypothesis that systematic hip strengthening programs may contribute to improvements in knee extensor mechanism alignment. However, considering the multifactorial nature of patellofemoral pain syndrome and the limited sample size of the present pilot study, these findings should be interpreted with caution and confirmed in larger, randomized, controlled trials.

### 4.4. Correlation Between Femoral Version, Tibial Torsion, and Patellar Alignment

The relationship between anatomical and functional factors has been extensively studied in recent years. Qiao et al. observed a correlation between increased tibial torsion, femoral anteversion, and lower-limb malalignment in patients with patellar instability [24]. Barton et al. also reported a significant association between elevated FNA and increased TT–TG distance in cases of patellar dislocation [56]. These findings suggest that structural anatomical characteristics may exacerbate patellofemoral stress patterns, while also highlighting the need for interventions that address not only bony morphology but also muscular control and functional alignment.

Recent musculoskeletal simulation models demonstrate that structural variations, such as femoral anteversion and tibial torsion, influence patellofemoral loading; however, adjustments in muscle activation may mitigate these effects [23]. This evidence supports the notion that selective hip strengthening may contribute to improvements in patellar alignment, even in individuals with anatomical predispositions.

### 4.5. Limitations

This study presents important limitations. The exercise protocol was applied to a small sample of only ten participants, as adherence was compromised by withdrawals related to the structured nature of the supervised exercise program. In addition, dynamic variables were not assessed since the computed tomography scans were limited to static analysis. Considering that small variations in femorotibial rotation may alter TT–TG distance [53], future studies should incorporate three-dimensional kinematic analyses to improve the understanding of these interactions.

Participants were instructed to maintain their usual daily activities and not to initiate any additional physical training or physiotherapy programs beyond the study protocol. However, physical activities performed outside the supervised environment were not monitored using specific instruments. The protocol consisted of 36 sessions; nevertheless, longer intervention periods may be required to evaluate potential structural bone adaptations, particularly in variables such as femoral neck anteversion (FNA) and TET. Additionally, although the study focused on tomographic measurements, the inclusion of pain scores and functional outcomes could provide a more comprehensive understanding of the clinical relevance of the observed changes.

Despite these limitations, as a pilot study evaluating the proposed strengthening protocol, the findings may serve as a foundation for future large-scale studies investigating the benefits of muscle strengthening programs in individuals with biomechanical lower-limb alignment alterations.

### 4.6. Perspective

It should be emphasized that only the TT–TG distance and Q angle demonstrated statistically significant changes, despite the small sample size and relatively small absolute mean differences observed (1.51 mm and 1.14°, respectively). These variations may seem subtle from a clinical perspective, but their detection was only possible through the use of high-precision three-dimensional computed tomography measurements, thus reinforcing the methodological value and sensitivity of the present study in identifying small changes in lower-limb alignment.

In contrast, no significant changes were observed in femoral neck anteversion (FNA) or tibial external torsion angle (TET), suggesting that these morphological characteristics may show limited responsiveness to conservative treatment. These findings are consistent with the previous literature emphasizing the importance of considering femoral anteversion and tibial torsion in the comprehensive evaluation of the lower limb. Furthermore, future studies should incorporate both morphological and functional variables, such as three-dimensional gait analysis, to improve the understanding of lower-limb biomechanics and support more accurate treatment selection [57].

Because patellofemoral pain syndrome (PFPS) may be associated with multiple morphological and functional factors, accurate diagnosis is essential for guiding targeted conservative treatment strategies. In the present study, the exercise protocol improved lower-limb alignment; however, it was not possible to determine whether strengthening of the hip or quadricep musculature contributed more substantially to this improvement. Consistent with the findings of Hott et al. [58], these results underscore the need for randomized clinical trials to determine which muscle group exerts the greatest influence on the conservative management of PFPS.

## 5. Conclusions

The strengthening program targeting the quadriceps and hip external rotator and hip abductor muscles promoted a reduction in knee valgus and improved axial lower-limb alignment, as evidenced by the significant reductions in Q angle and TT–TG distance. However, no significant changes were observed in structural variables such as femoral neck anteversion (FNA) and tibial external torsion angle (TET).

The findings obtained, even in a small sample, particularly regarding TT–TG distance, highlight the importance of using more robust assessment methods capable of analyzing rotational characteristics of the skeletal structure in the axial plane. In this context, proximal muscle strengthening appears to be an important, non-invasive strategy for the treatment of individuals with patellofemoral pain syndrome.

## Figures and Tables

**Table 1:** Physical training program for strengthening the quadriceps femoris muscles, external rotator, and hip abductor muscles. The letters A–L represent the order in which the exercises were performed and correspond to each exercise shown in Figure 1.

Exercises		Contraction	Equipment	Number Sets	Number of Reps/Time	Training Load
A	Hip abduction	Isometric	Against the wall	4	40 s	Bodyweight only
B	Knee extension	Isotonic	Leg extension machine	3	20	60% of 1 RM
					15	70% of 1 RM
					10	70% of 1 RM
C	Hip abduction	Isotonic	Hip abduction machine	3	20	60% of 1 RM
					15	70% of 1 RM
					10	70% of 1 RM
D	Squat with hip abduction and proprioceptive control	Isotonic	Resistance bands	3	15	1.3–1.7 kgf
E	Abduction with external rotation	Isotonic	Resistance bands	3	20	0.7–1.0 kgf
					15	1.3–1.7 kgf
					10	2.0–2.6 kgf
F	Alternating single-leg hip bridge	Isometric	None	6	30 s	Bodyweight only
G	Quadruped hip abduction with external rotation	Isotonic	Resistance bands	3	20	0.7–1.0 kgf
					15	1.3–1.7 kgf
					10	2.0–2.6 kgf
H	Hip abduction with leg extension	Isotonic	Ankle resistance weights	3	15	2 or 3 kg
					15	3 or 4 kg
					15	4 or 5kg
I	Lateral band walk	Isotonic	Resistance bands	3	20 steps	0.7–1.0 kgf
						1.3–1.7 kgf
						2.0–2.6 kgf
J	Seated hip external rotation	Isotonic	Cable machine using a low pulley attachment	3	20	60% of 1 RM
					15	70% of 1 RM
					10	70% of 1 RM
K	Hip bridge with proprioceptive control in abduction	Isometric	Resistance bands	3	40 s	1.3–1.7 kgf
L	Hip abduction combined with external leg rotation	Isotonic	Cable machine using a low pulley attachment	3	20	60% of 1 RM
					15	70% of 1 RM
					10	70% of 1 RM

(kg) kilograms; (kgf) kilogram-force; (1 RM) one-repetition maximum; (s) seconds.

## Data Availability

The original contributions presented in the study are included in the article, further inquiries can be directed to the corresponding author.

## Abbreviations

The following abbreviations are used in this manuscript:

BMI	body mass index
TT–TG	tibial tubercle–trochlear groove distance
FNA	femoral neck anteversion angle
TET	tibial external torsion angle
PFPS	patellofemoral pain syndrome
3D	three-dimensional
CT	computed tomography
VAS	visual analog scale
FH	fibular head
ANOVA	analysis of variance
VMO	vastus medialis oblique
VL	vastus lateralis
